# External Radiotherapy in Mozambique: Report of the First Five Years of Activity of the Radiotherapy Service of the Maputo Central Hospital

**DOI:** 10.1002/cam4.71199

**Published:** 2025-09-02

**Authors:** Filipa Fontes, Alberto Gudo Morais, Joaquina Nhampule, Sara Navarro, Narciso Sitoe, Leonel Amisse, Satish Tulsidás, Cesaltina Lorenzoni, Carla Carrilho, Nuno Lunet

**Affiliations:** ^1^ Abordagem de Lesões Pré‐Cancerosas e Cancro Precoce – Centro de Investigação (CI‐IPOP), Instituto Português de Oncologia do Porto (IPO‐Porto) & Porto Comprehensive Cancer Center (Porto.CCC) & RISE@CI‐IPOP (Rede de Investigação Em Saúde) Porto Portugal; ^2^ Departamento de Ciências da Saúde Pública e Forenses e Educação Médica, Faculdade de Medicina Universidade do Porto Porto Portugal; ^3^ Serviço de Radioterapia, Hospital Central de Maputo Maputo Mozambique; ^4^ Departament of Clinical Training & Education Mercurius Health S.A. Taguspark, Núcleo Central Expansão Porto Salvo Portugal; ^5^ Serviço de Oncologia Médica Hospital Central de Maputo Maputo Mozambique; ^6^ Serviço de Anatomia Patológica Hospital Central de Maputo Maputo Mozambique; ^7^ Departamento de Patologia, Faculdade de Medicina Universidade Eduardo Mondlane Maputo Mozambique; ^8^ EPIUnit Instituto de Saúde Pública da Universidade do Porto & Laboratório Para a Investigação Integrativa e Translacional Em Saúde Populacional (ITR) Porto Portugal

**Keywords:** developing countries, healthcare, neoplasm, radiotherapy

## Abstract

**Background:**

A Radiotherapy Service (RS) started working in the Maputo Central Hospital (MCH), Mozambique, in August 2019. Here we describe its first 5 years of activity.

**Methods:**

A total of 810 patients who underwent external radiotherapy between August 2019 and December 2023 were considered for the analysis. Sociodemographic and clinical data were retrieved from the RS database, and the MCH Cancer Registry was used to obtain cancer incidence data.

**Results:**

There was an increase in newly admitted patients each year, except in 2023 (161 in 2020, 188 in 2021, 238 in 2022, and 185 in 2023). Patients admitted in 2019 were more likely to be from Maputo city and to receive palliative radiotherapy. The proportion of patients starting radiotherapy within 12, 24, 36, and 48 months after diagnosis was 38.8%, 78.1%, 91.7%, and 96.6%, respectively. There was an increasing trend (*p* for trend < 0.001) in the proportion of patients admitted with cervical cancer over time (from 34.2% in 2019 to 46.0% in 2023). Differences in radiotherapy initiation within 12 months were observed according to treatment intent (42.6% for palliative vs. 36.9% for curative; *p* = 0.014) and cancer site (64.5% for rectal/anal cancer vs. 34.8%, 29.5%, and 37.0% for cervical, breast and prostate cancer patients, respectively; *p* < 0.001).

**Conclusions:**

The RS‐MCH improved access to radiotherapy for cancer patients, particularly those with cervical and breast cancers. Patients undergoing palliative treatment and those with anal/rectal cancers started radiotherapy earlier. Further research is needed to evaluate the impact of radiotherapy on mortality, survival, and in patient‐centered outcomes.

## 1 Introduction

Along with surgery and chemotherapy, radiotherapy is recognized as an essential component of cancer treatment. It is estimated that, alone or combined with other options of cancer management, radiotherapy is indicated for approximately 60% of cancer patients during the course of their disease [1]. In 2017, the World Health Organization included radiotherapy in a list of basic and priority medical devices required for management of cancer [2], with the main goal of increasing its availability in low‐ and middle‐income countries.

It is estimated that a yearly number of around 2.4 million new cancer cases and 1.6 million cancer deaths will take place in Africa by 2045, reflecting an increase of more than 100% in relation to the GLOBOCAN estimates for 2022 [3]. In addition to earlier detection, it is paramount to enhance cancer treatment in Africa to improve the quality of life and survival of patients in this context. Despite the increase in the availability of external beam radiotherapy since 2012 (from 43% to 52% of the countries in 2020), approximately half of the radiotherapy units remained concentrated in two countries (Egypt and South Africa) [4].

In Mozambique, a radiotherapy unit was installed in the 1960s and operated until 1996. Since then, patients requiring radiotherapy have been referred to other countries, such as South Africa, India, and Portugal [5]. The initiative to establish a new radiotherapy unit began in 2006 with the adherence of Mozambique to the International Atomic Energy Agency, the creation of a National Authority for Atomic Energy, and the implementation of training programs for health professionals. As a result of this process, a radiotherapy unit was implemented at the Maputo Central Hospital (MCH), the national reference for cancer treatment, and has been operating since August 2019. Therefore, we aim to describe the activity of the Radiotherapy Service (RS) of the MCH since its onset, over the first 5‐year period (2019–2023).

## 2 Methods

The RS at MCH is equipped with a linear accelerator (Elekta Synergy Platform) and a computed tomography machine (Siemens SOMATOM Perspective) for radiotherapy treatment simulations, both of which operate every weekday from 7:30 a.m. to 4:00 p.m. The radiotherapy team is composed of three radiation oncologists, three medical physicists, nine technicians, three radiotherapy nurses, one social worker, and three administrative staff members.

Patients proposed for radiotherapy are usually referred to the RS by the Oncology Service. In some cases, patients come directly from other MCH departments after a multidisciplinary team discussion, aiming to expedite radiotherapy simulation and other preparatory procedures. Patients from private clinics are referred through *Clínica Especial*, a specific section of the hospital dedicated to private healthcare.

All patients submitted to radiotherapy in the RS between August 2019 and December 2023 were considered for the present analysis. Data on sociodemographic (sex, age, place of residence), clinical (cancer site), and characteristics related to the radiotherapy treatment (intent of the treatment and start date of treatment) were retrieved from a database from the RS. The MCH Cancer Registry was used to obtain cancer incidence data. It was defined according to the recommendations of the International Agency for Research on Cancer, the International Association of Cancer Registries, and the European Network of Cancer Registries [6].

Patients' characteristics are presented as counts and proportions, overall and for each calendar year, and compared using the chi‐squared test and the Wilcoxon test for trend. The number of patients admitted to the RS was plotted by year and month. The time (in months) from incidence to the beginning of radiotherapy—overall, by treatment intent, and by cancer site—was plotted (1‐Kaplan–Meier) and compared using the log‐rank test. Additionally, the medians and percentiles 25 (P25) and 75 (P75) are reported.

## 3 Results

**FIGURE 1:**
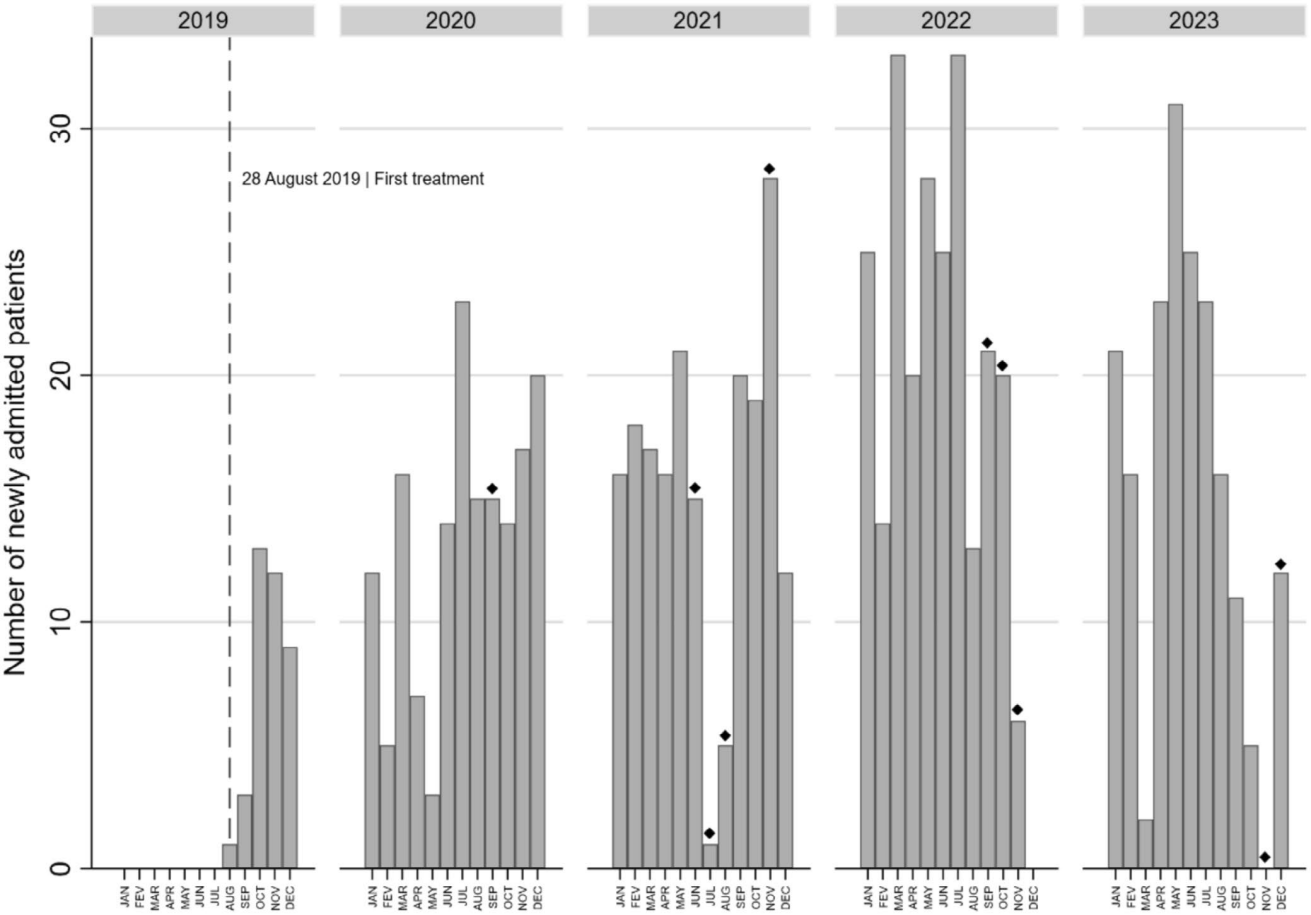
Number of patients newly admitted to the Radiotherapy Department of the Maputo Central Hospital (Mozambique), by month of admission. Diamonds represent months with at least five working days of complete or partial interruption of treatments due to equipment breakdowns or maintenance.

Between August 2019 and December 2023, a total of 810 patients underwent external radiotherapy at the RS of the HCM, all treated on an outpatient basis. Overall, there was a consistent trend of an increasing number of newly admitted patients each year, except for 2023 (Table 1, Figure 1). Since its inception, there have been 10 months with at least five working days of complete or partial interruption in treatments due to equipment breakdowns or maintenance.

**TABLE 1:** Sociodemographic and clinical characteristics of patients newly admitted to the Radiotherapy Unit of the Maputo Central Hospital (2019–2023).

		Total [*N* = 810]	2019 [*N* = 38]	2020 [*N* = 161]	2021 [*N* = 188]	2022 [*N* = 238]	2023 [*N* = 185]	*p*
Sex								
	Male	150 (18.5)	10 (26.3)	40 (24.8)	36 (19.2)	35 (14.7)	29 (15.7)	
	Female	660 (81.5)	28 (73.7)	121 (75.2)	152 (80.8)	203 (85.3)	156 (84.3)	0.058^c^
Age (years) [*N* = 804]								
	< 45	306 (38.1)	19 (50.0)	57 (35.4)	76 (40.4)	91 (38.2)	63 (35.2)	
	45–55	233 (29.0)	5 (13.2)	48 (29.8)	52 (27.7)	72 (30.3)	56 (31.3)	
	> 55	265 (33.0)	14 (36.8)	56 (34.8)	60 (31.9)	75 (31.5)	60 (33.5)	0.553^d^
Province of residency [*N* = 796]								
	Maputo city	271 (34.0)	19 (50.0)	63 (39.1)	52 (27.7)	86 (36.1)	51 (29.8)	
	Maputo	275 (34.5)	5 (13.2)	41 (25.5)	64 (34.0)	84 (35.3)	81 (47.4)	
	Gaza	62 (7.8)	4 (10.5)	13 (8.1)	11 (5.8)	24 (10.1)	10 (5.8)	
	Inhambane	48 (6.0)	1 (2.6)	12 (7.4)	15 (8.0)	7 (2.9)	13 (7.6)	
	Sofala	38 (4.8)	0 (0.0)	7 (4.4)	12 (6.4)	13 (5.5)	6 (3.5)	
	Other provinces	98 (12.3)	9 (23.7)	23 (14.3)	32 (17.0)	24 (10.1)	10 (5.8)	
	Other countries	4 (0.5)	0 (0.0)	2 (1.2)	2 (1.1)	0 (0.0)	0 (0.0)	< 0.001^e^
Intent of treatment [*N* = 809]								
	Curative	553 (68.4)	20 (52.6)	106 (65.8)	121 (64.4)	188 (79.0)	118 (64.1)	
	Palliative	256 (31.6)	18 (47.4)	55 (34.2)	67 (35.6)	50 (21.0)	66 (35.9)	< 0.001^f^
Cancer site [*N* = 807]^a^								
	Cervix uteri	358 (44.4)	13 (34.2)	54 (34.0)	84 (44.9)	122 (51.3)	85 (46.0)	
	Breast	183 (22.7)	9 (23.7)	39 (24.5)	35 (18.7)	53 (22.3)	47 (25.4)	
	Rectum and anus	33 (4.1)	2 (5.3)	10 (6.3)	5 (2.7)	7 (2.9)	9 (4.9)	
	Prostate	30 (3.7)	1 (2.6)	6 (3.8)	8 (4.3)	8 (3.4)	7 (3.8)	
	Others	176 (21.8)	11 (29.0)	40 (25.2)	45 (24.1)	43 (18.1)	37 (20.0)	
	Uknown^b^	27 (3.4)	2 (5.3)	10 (6.3)	10 (5.4)	5 (2.1)	0 (0.0)	0.044^g^

^a^ Excluded three patients submitted to radiotherapy due to benign conditions.

^b^ For 31 patients, information on cancer site was not available in the database from the Radiotherapy Unit, and information was not possible to obtain from Maputo Central Hospital Cancer Registry.

^c^ *p*‐value for trend: 0.006.

^d^ *p*‐value for trend: 0.771.

^e^ *p*‐value for trend: 0.455.

^f^ *p*‐value for trend: 0.098.

^g^ *p*‐value for trend: < 0.001.

Most of the patients admitted were females (81.5%), and more than two‐thirds were dwellers in Maputo city or Maputo province. Patients with cervical and breast cancer accounted for 44.4% and 22.7% of the total, respectively, and nearly one‐third were admitted with a palliative intent. Differences were noted in the patients' province of residence, treatment intent, and cancer site across the years. Those admitted in 2019 were more likely to be from Maputo city (50.0% compared to 39.1%, 27.7%, 36.1%, and 29.8% in subsequent years; *p* < 0.001) and more frequently received palliative radiotherapy (47.4% in 2019 vs. 34.2%, 35.6%, 21.0%, and 35.9% in subsequent years; *p* < 0.001). There was a tendency for an increasing proportion of newly admitted patients with cervical cancer over time (34.2%, 34.0%, 44.9%, 51.3%, and 46.0% in 2019, 2020, 2021, 2022, and 2023, respectively; *p*‐value for trend < 0.001) (Table 1).

**FIGURE 2:**
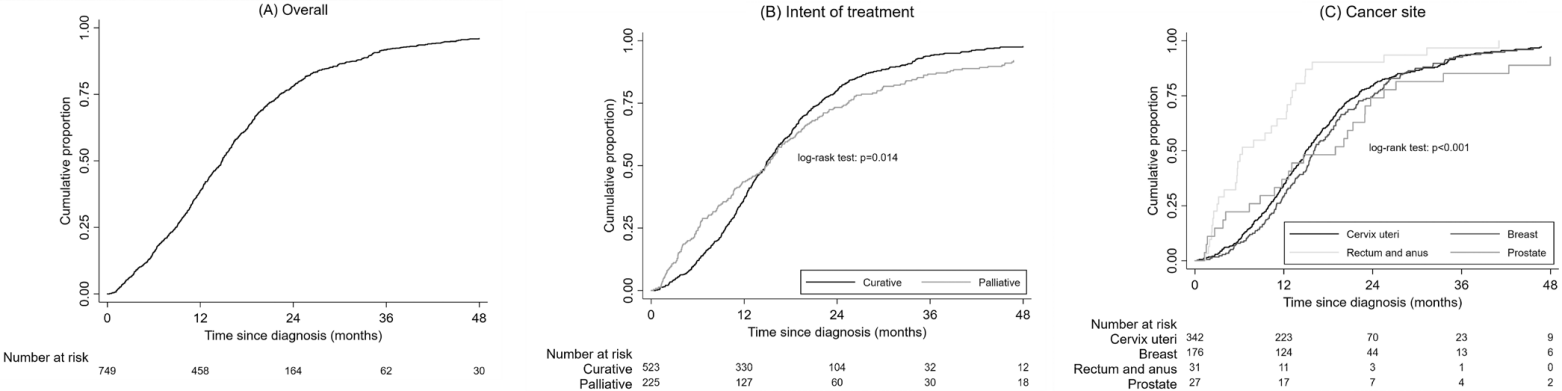
Cumulative incidence estimates of time (months) from diagnosis to radiotherapy initiation, in overall (A) and stratified by intent of treatment (B) and cancer site (C).

The median time (P25—P75) from diagnosis to radiotherapy initiation was 37.6 (22.4–56.4) months. The proportion of patients who started radiotherapy within 12, 24, 36, and 48 months after cancer diagnosis was 38.8%, 78.1%, 91.7%, and 96.6%, respectively (Figure 2A). At 12 months, 36.9% of patients proposed for curative treatment and 43.6% of those proposed for palliative treatment had started radiotherapy, while the cumulative proportion of patients receiving curative treatment who had initiated radiotherapy was higher at 36 months (93.9% vs. 86.7%; *p* = 0.014). Furthermore, the cumulative proportion of patients who initiated radiotherapy within 12 months after diagnosis was higher among those with rectal/anal cancer (64.5% compared to 34.8%, 29.5%, and 37.0% in for cervical, breast and prostate cancer, respectively; *p* < 0.001).

## 4 Discussion

The initial increase in the number of patients treated aligns with the implementation of a new service. However, periods of maintenance or breakdown of the equipment may have contributed to limiting the increase in the number of cases treated over time. Additionally, although the first case of COVID‐19 in Mozambique was reported on March 2020 [7], and the RS continued to operate during the pandemic, we can not exclude the possibility that the pandemic had some impact on demand or caused interruptions of the planned care, influencing the number of newly admitted patients. The decrease in new admissions observed in 2023 may be partially explained by the increasing proportion of patients undergoing curative treatment, which occupies the equipment for longer periods than palliative care, reducing the turnover and limiting the availability of vacancies for new patients.

The higher proportion of patients treated with a palliative intent in 2019, when compared to the subsequent years, could reflect both an initial focus on managing advanced‐stage cancers during the early months of the radiotherapy unit's operation, but also a tendency for early stages at diagnosis across time. The latter trend was previously reported for cervical cancers diagnosed in the period prior to the radiotherapy unit inception [8]. The gradual increase in the number of cervical cancer cases admitted over time may reflect the increase in the capacity to treat more cases, but also an increasing detection of the disease, as well as improvements in referral systems. In fact, a multidisciplinary tumor board of the Gynecology and Obstetrics Department was implemented in 2016 and may have contributed to the progressive improvement in the referral of patients to perform radiotherapy.

During the first 12 months after diagnosis, a larger proportion of patients had started palliative radiotherapy compared to those receiving curative therapy. However, after this period, the trend was reversed. The earlier start in palliative cases can be explained by the urgent nature of palliative care, where the primary goal is to relieve symptoms and improve quality of life. In contrast, curative treatment often involves a combination of surgery, chemotherapy, and radiotherapy, and therefore radiotherapy may not be the first‐line treatment after diagnosis, which could, at least in part, explain the trend observed. Significant variation in radiotherapy timing is also observed across different cancer types. Within the first 12 months, a higher proportion of rectal/anal cancer patients began radiotherapy compared to patients with cervical, breast, and prostate cancers. This may be attributed to differences in treatment intent, but also to different clinical guidelines for the treatment of each cancer type. For instance, rectal and anal cancers often require prompt radiotherapy as part of multimodal treatment approaches [9, 10], whereas for cancers like breast and prostate, radiotherapy might be scheduled later depending on individual treatment pathways, particularly in the context of hormonal therapies or surgery [11, 12, 13]. Consistent with this, delays in the initiation of radiotherapy may be at least partially explained by the initial use of other cancer treatment modalities, as most patients were admitted with a curative intent and therefore were likely to have been previously submitted to surgery and/or chemotherapy, but also by the limited capacity of the RS.

Patients with cervical and breast cancers comprised two‐thirds of the treated cases. Survival rates are available for patients diagnosed with cervical (2016–2018) and breast cancers (2016–2017), prior to the establishment of the radiotherapy unit and followed at MCH [8, 14]. The reported overall survival rate for cervical cancer was 51.0% at 2 years [8], while for breast cancer it was 62.6% at 3 years [14]. Further research is needed to evaluate the impact of the introduction of radiotherapy at MCH on mortality and survival rates and in patient‐centered outcomes.

This study provides, for the first time, an overview of the activity of the RS of the MCH. However, some limitations must be acknowledged. The absence of national data estimating the number of cancer patients requiring radiotherapy precluded the estimation of the proportion of patients benefiting from it. Nevertheless, as the RS is still in its early years of operation and currently treating patients diagnosed in previous years, any utilization rate calculated would likely not accurately reflect its current capacity. Also, the inclusion of other clinical information, such as cancer stage at diagnosis, cancer treatment options, and comorbidities, could have enhanced the interpretation of our results. In conclusion, the establishment of a RS at MCH significantly enhanced access to radiotherapy for cancer patients in Mozambique, particularly for those with cervical and breast cancers. Patients proposed for palliative treatment and those with anal and rectal cancers started radiotherapy earlier. Despite an increase in patient admissions over the study period, challenges remain, including operational disruptions due to equipment breakdown or maintenance.

## Author Contributions

**Filipa Fontes:** writing – review and editing, formal analysis, methodology, writing – original draft. **Alberto Gudo Morais:** data curation, writing – review and editing. **Joaquina Nhampule:** data curation, writing – review and editing. **Sara Navarro:** data curation, writing – review and editing. **Narciso Sitoe:** data curation, writing – review and editing. **Leonel Amisse:** data curation, writing – review and editing. **Satish Tulsidás:** writing – review and editing. **Cesaltina Lorenzoni:** writing – review and editing. **Carla Carrilho:** conceptualization, data curation, funding acquisition, writing – review and editing, methodology. **Nuno Lunet:** conceptualization, writing – review and editing, methodology.

## Ethics Statement

The study was approved by the Joint Institutional Bioethical Committee of Faculty of Medicine of Eduardo Mondlane University and Maputo Central Hospital (reference No. CIBS FM&HCM/71/2017). The Informed consent was waived because of the retrospective nature of the study.

## Conflicts of Interest

The authors declare no conflicts of interest.

## Data Availability

The data used in this article will be shared upon reasonable request to the corresponding author.
